# Supplementary material for: Noise reduction in X-ray photon correlation spectroscopy with convolutional neural networks encoder–decoder models
Source: Sci Rep. 2021 Jul 20;11:14756. doi: 10.1038/s41598-021-93747-y (PMC8292438; doi:10.1038/s41598-021-93747-y)
Supplement: Supplementary file 1 — Supplementary Information. [file 41598_2021_93747_MOESM1_ESM.pdf]

Supplemental Materials for: Noise Reduction in X-ray Photon Correlation Spectroscopy with Convolutional Neural Networks Encoder-Decoder Models

Tatiana Konstantinova[1], Lutz Wiegart[1], Maksim Rakitin[1], Anthony M. DeGennaro[1,+], and Andi M. Barbour[1,*]

[1]Brookhaven National Laboratory, NSLS-II, Upton, NY 11973, USA
[+]adegennaro@bnl.gov
[*]abarbour@bnl.gov

I. Distribution of dynamics parameters for the training, validation and test set

Training and validation sets. The distributions of the dynamics parameters for the train and validation sets are obtained by fitting the full-range 1TCF (calculated from all available frames) for each ROI of each experiment to the Eq. 3 and by sampling according to the number of inputs obtained from the corresponding 2TCF. For example, for an experimental dataset with 5 ROIs and 200 frames, each ROI gives seven 50×50 inputs. We obtain 5 sets of parameters (β, Γ, α and C_∞), one for each ROI. We then copy each parameter set to the training parameters pool 7 times. The distributions of the parameters in the training and the validation pools are shown in Fig. S1.

Test set. The test set contains data from similar experiments as used for training/validation. Each experiment has 600-1000 frames. This set is used for establishing applicability limits of the model. While the 1TCFs for these data are not perfectly described by Eq. 3, they can be approximated with it. We obtain the distributions for the dynamics parameters in a similar way as for the training/validation data. The distributions are shown in Fig. S2.

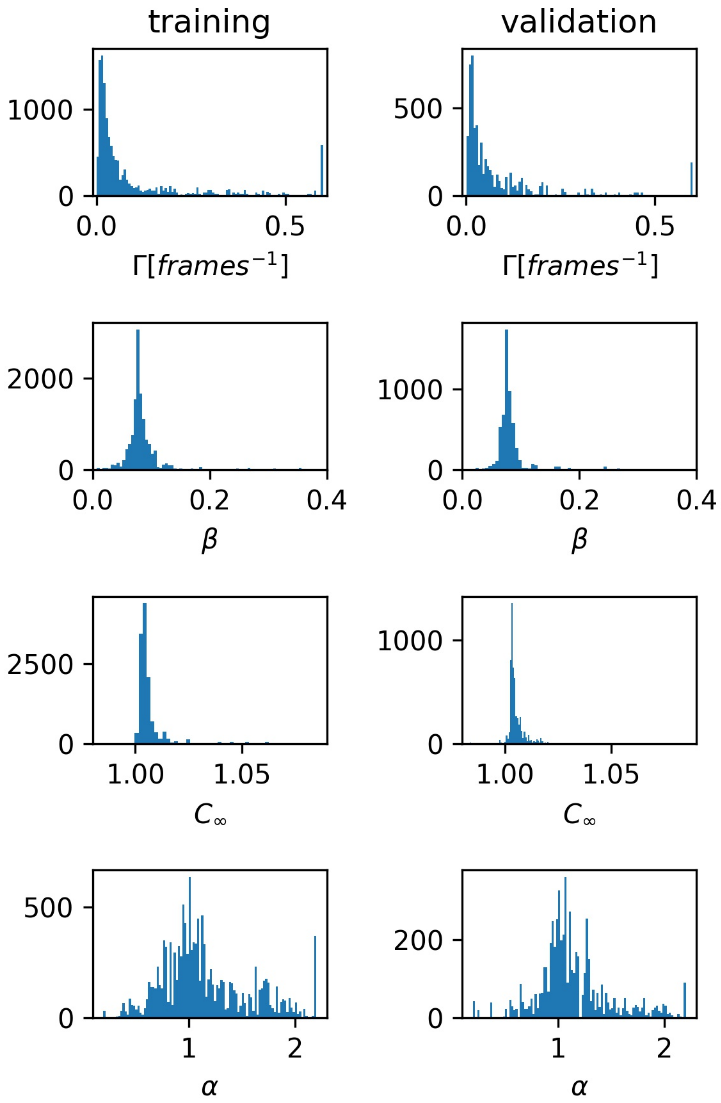

Figure S1. Dynamics' parameters distribution for the training (left column) and validation (right column) sets.

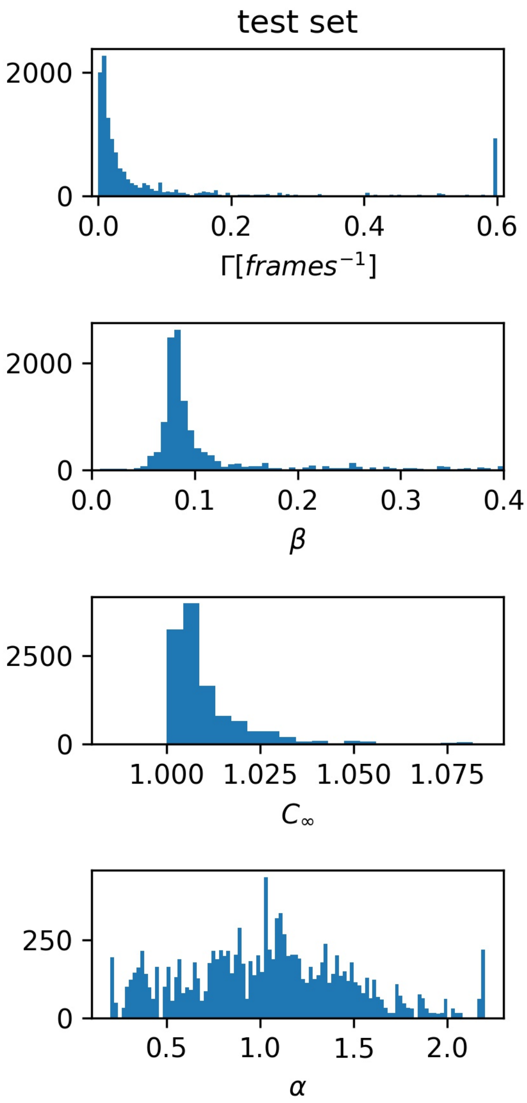

Figure S2. Dynamics' parameters distribution for the test set.

II. Model performance on the validation set

We check the performance of the best ensemble (10 models) on the validation set by comparing the errors of dynamics parameters (Eq. 3) extracted from the raw input 2TCFs and the denoised outputs. The error are calculated with respect to the corresponding parameters, extracted from the raw full-range (target) 2TCF. The comparison is shown in Fig. S3. One can see that the rate Γ is extracted from the ensemble's output with a good precision for $\Gamma < 0.2 frames^{-1}$ (the contrast drops by half in 2 or more frames). Above $\Gamma = 0.2 frames^{-1}$, the variance for the Γ extracted from the denoised data is similar to the one of the Γ extracted from the raw data. Other dynamics parameters are generally extracted with better precision from the denoised data than from the raw data. Moreover, for some of the raw inputs, the fit to the Eq. 3 does not converge within the reasonable parameters bounds, but the corresponding model outputs can be fit within the same bounds. Note, the precision of the β is largely dependent on the accuracy of extracting the speckle visibility from the photon distribution in a single frame, which serves as the normalization parameter.

The comparison is summarized in Table S1. It includes the mean square error (MSE) of 1TCF, the mean absolute relative error (MRE) for the Γ and the MSE for all other parameters. It is important to consider the relative errors for the Γ instead of absolute errors since its values can be very close to zero and a small absolute error may still be significant.

Note, that performance of the model is optimized to the validation set via the early stopping criteria and that the good accuracy is expected for the set. The model's generalizability and application limits are determined below based on the test examples, which are not accessed during the training.

	raw 50×50	model output
do not converge	379	0
MSE(1TCF)	2.8e-5	1.05e-5
MRE Γ	0.72	0.46
MSE β	0.0045	0.0016
MSE C_∞	0.00027	0.00009
MSE α	0.45	0.16

Table S1. Comparison of parameters' errors extracted from the raw input data and from the corresponding model's output for the validation set (5449 examples).

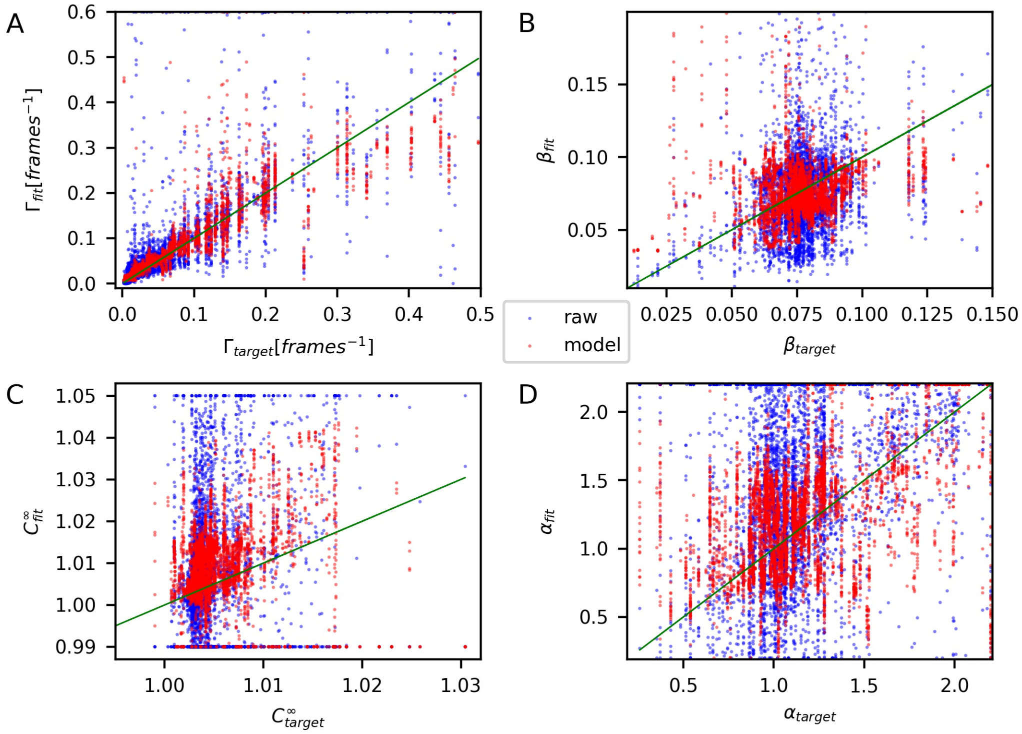

Figure S3. Comparison of dynamics parameters extracted for raw (blue) and denoised (red) 50×50 2TCF from the validation set. Horizontal axes reflects the values extracted from the corresponding target 2TCF. The green lines with slope 1 are included for convenience to represent a full correlation between the target and the fit.

III. Model performance on the test set

We check the performance of the best ensemble (10 models) on the test set by comparing the errors of dynamics parameters (Eq. 3) extracted from the raw input 2TCFs and the denoised outputs with respect to the corresponding parameters extracted from the raw full-range (target) 2TCF. Unlike the training/validation sets that only include the 2TCFs with noticeable de-correlation within the first 50 frames, the test set includes all available 2TCFs. There are several difficulties for comparison when all four parameters in the Eq. 3 are relaxed:

- Sample dynamics are more complicated than the Eq. 3 and may contain several exponential terms and/or terms with variable parameters.
- The considered 49 time points might not be enough to unequivocally distinguish between two or more possible dynamics with different parameters, especially in cases of very slow dynamics.

The goal of this work is not to correctly characterize dynamics of the materials used for experiments in the test set, but to identify how close the outputs of the model are to the respective correlation functions measured with good statistics (the target). Thus, for a more deterministic comparison, we reduce the number of parameters and fix α to 1. Fixing or highly restricting the fit parameters is a common practice in XPCS analysis. The results are shown in Fig. S4.

Firstly, we set the applicability limits of the model by identifying the regions where the relative error for Γ is too large. The distribution of relative errors of Γ for different target values of Γ_{target} indicates that the ensemble does not perform well for small values of $\Gamma < 0.01 frames^{-1}$. At such rates, only a portion of the dynamics is complete within the first 50 frames: the contrast drops by 60 or less percent at furthest available (50th) frame. Above $\Gamma = 0.01 frames^{-1}$ the relative error of Γ is generally below 100%, which can be an acceptable level depending on the experiment. We thus select $\Gamma = 0.01 frames^{-1}$ as the lower bound for the model applicability range. We select $\Gamma = 0.15 frames^{-1}$ as the upper bound since above this value a better precision can be achieved from the raw outputs than from the model. The upper bound is smaller than for the validation set, which partially can be explained by errors in estimation of contrast used for the input normalization. For the test set, the normalization parameter is estimated from the first off-diagonal elements of the input 50×50 2TCF, while for the training and validation sets it is estimated from all available single frames using speckle visibility spectroscopy. Errors in contrast estimation can be especially pronounced for the cases of fast dynamics, where the contrast drops significantly within a a single frame.

The comparative accuracy for the other dynamics parameters are also shown in Fig. S4. The accuracy for measuring amplitude β is similar for the raw data and for the model output, which is expected since the normalization for the model inputs is based on the contrast measured for the raw data. However, the baseline is under-fit for the large values, that are not present enough in the training set. For comparison, Figure S5 shows the same dependencies for the case when the α is not fixed.

The summary statistics for the Fig. S4 and Fig. S5 are in the Table S2. Only the cases with $0.01 frames^{-1} < \Gamma_{target} < 0.15 frames^{-1}$ are included.

	raw 50×50	model output
MSE(1TCF)	0.012	6.6e-5
MRE Γ, $\alpha = 1$	0.56	0.28
MSE β, $\alpha = 1$	0.001	0.0007
MSE C_∞, $\alpha = 1$	0.0007	0.00065
do not converge, $\alpha = 1$	415	18
MRE Γ	0.76	0.33
MSE β	0.0042	0.0011
MSE C_∞	0.0006	0.0002
MSE α	0.69	0.24
do not converge	343	6

Table S2. Comparison of parameters errors extracted from the raw test data (7228 examples) and from the corresponding model outputs.

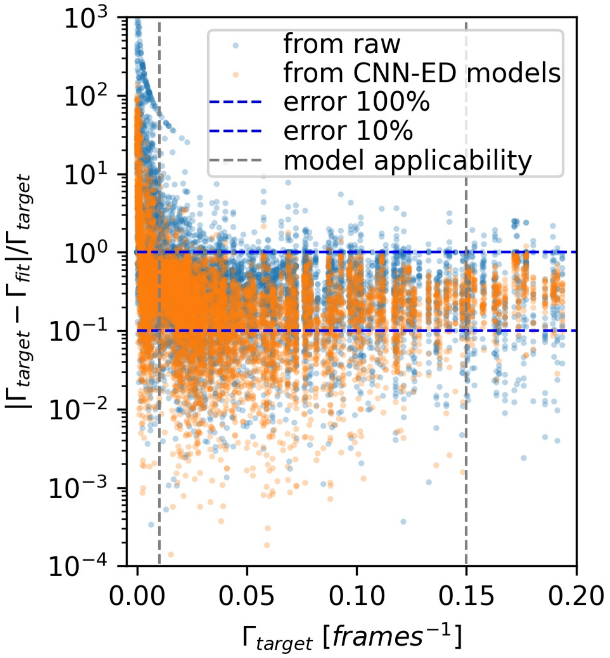

Figure S4. Comparison of accuracy of extracting the dynamics' parameters with $\alpha = 1$ for raw (blue) and denoised (orange) 50×50 2TCFs from the test set. The left panel shows the relative error of Γ versus underlying values of Γ_{target} (extracted from full-sized 2TCF). The vertical dashed lines show the lower and the upper boundaries of the model applicability. The right panel show the values for each of the dynamics parameters for the inputs within the model applicability limits versus the values extracted from the corresponding target 2TCF.

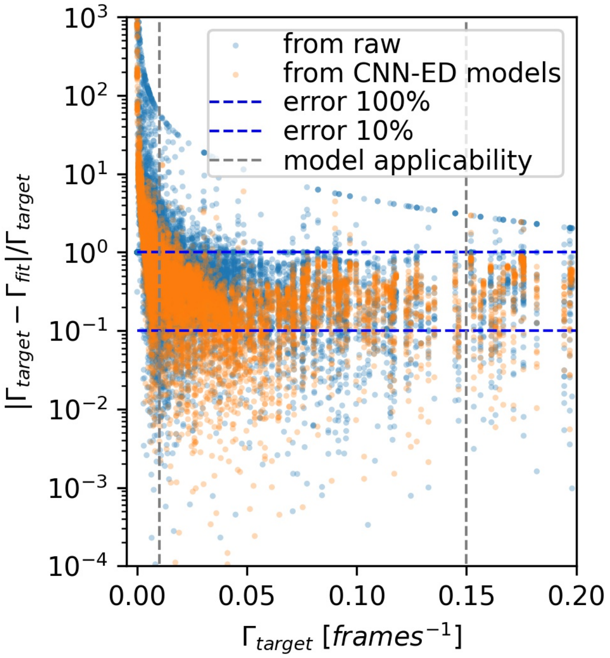

Figure S5. Comparison of accuracy of extracting the dynamics' parameters with the relaxed parameter α for raw (blue) and denoised (orange) 50×50 2TCFs from the test set. The left panel shows the relative error of Γ versus underlying values of Γ_{target} (extracted from full-sized 2TCF). The vertical dashed lines show the lower and the upper boundaries of the model applicability. The right panel show the values for each of the dynamics parameters for the inputs within the model applicability limits versus the values extracted from the corresponding target 2TCF.

IV. Model performance at different noise levels

Above, we have identified the limiting cases of rates of sample dynamics, beyond which the model does not perform well. It is expected that the noise level of input 2TCFs can limit the model accuracy as well. As the measure of noise we use the average standard deviation of pixel values within 5×5 fields of an input 2TCF. To calculate the noise, a 2TCF image is split into 5×5 pixels regions, and a standard deviation of intensity is calculated for each region. Then, the values are averaged among all the regions. The values are calculated prior to normalization of contrast to 1 (typical contrast values are 0.06-0.1). We compare $MRE(\Gamma)$ for the raw input data and the model outputs from the Fig. S5 as functions of the input noise. The comparison is shown in Fig. S6. Only the examples within the $0.01 frames^{-1} < \Gamma_{target} < 0.15 frames^{-1}$ are included. The accuracy of parameters extracted from both the raw data and from the model output deteriorates at larger noise. However, in a wide range of input noise the accuracy of the model's output is better than the raw 2TCF values as it does not have any extreme errors. Above a certain noise level (around 0.2), the $MRE(\Gamma)$ is comparable for both model's output and the raw data.

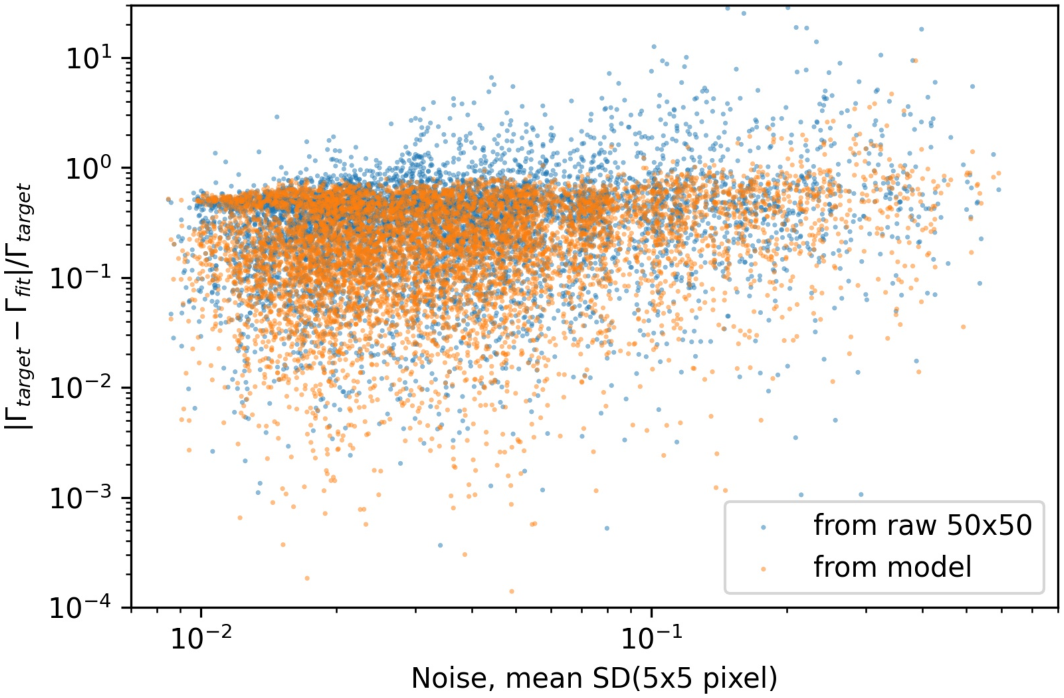

Figure S6. Relative error of Γ as a function of 2TCF noise.

The relative error of Γ extracted from the model outputs is shown in Fig. S7 as a function of the underlying rate of sample's dynamics Γ_{target} (as in Fig. S4) and the noise level of the input 2TCF. All tests examples are included.

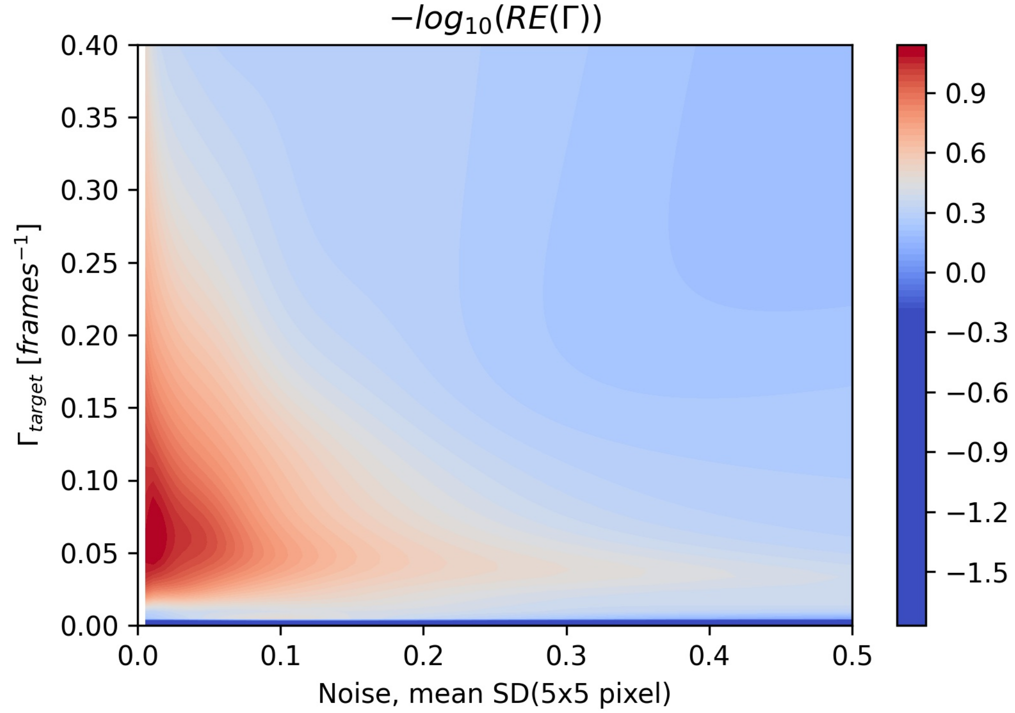

Figure S7. The contour plot of the logarithm of relative error for Γ as a function of underlying values of Γ_{target} and the noise level in 2TCF (the mean of standard deviations withing 5×5 pixel fields). High precision (low error) is observed for low-noise inputs for dynamics with $0.01 frames^{-1} < \Gamma < 0.15 frames^{-1}$ and low precision is observed for inputs with large noise and/or extreme dynamics rates. A log-normal kernel smoothing is used for interpolation and reducing the scatter.

V. Analysis of CNN-ED layers

To better understand how a CNN-ED model handles different levels of noise, we look at the activation maps of its layers for inputs with low (Fig. S8) and high (Fig. S9) noise. For the case of the low noise input, not all channels in the encoder layers are activated. However, for the case of the high noise input, all channels are activated, meaning that some channels are meant for handling the extreme pixel values. The latent space variables are generally non-zero for all types of inputs.

Due to the nature of the 2TCF and the scaling of the inputs, the "good" values of the inputs and outputs should be between 1 and 2. To see how different pixel values are transformed during the encoding stage, we look (Fig. S10) at outputs of the second encoding layer after application of the *ReLU* function. Models trained with different random weight initialization have different nonlinear activation functions. The activation functions of the encoder for the model in Fig. S8 and Fig. S9 are shown in Fig. S10. One can see that some layers (e.g. 5, 8, 9) are only activated at small (negative) correlation values, which are the noise since they are unnatural for the correlation function according to the Eq. 1.

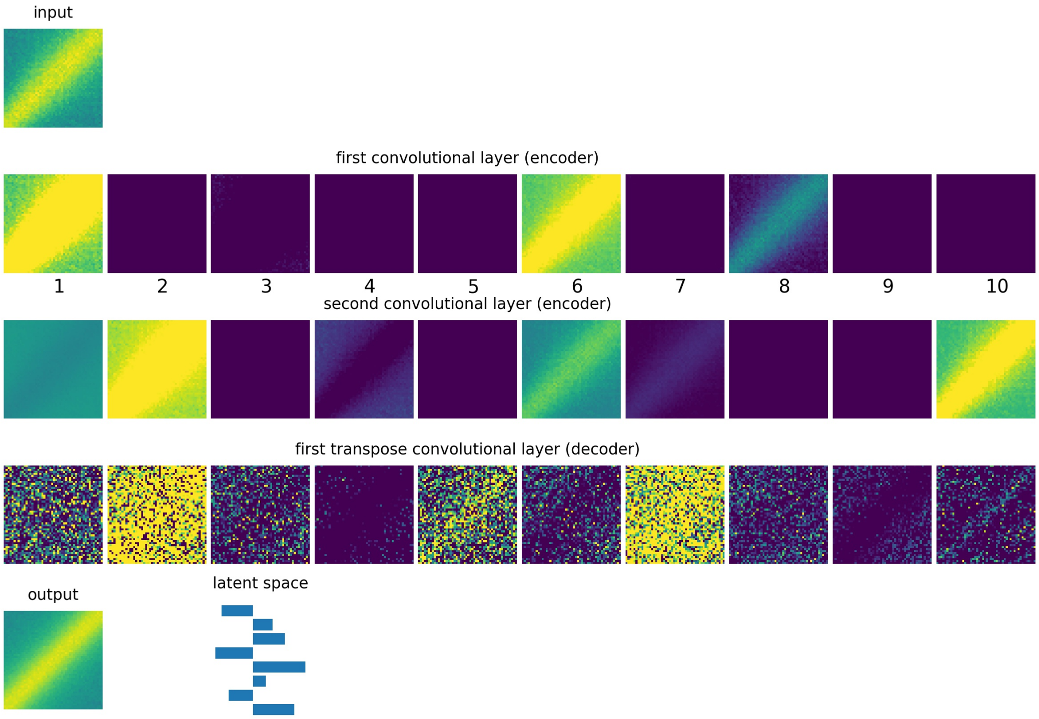

Figure S8. Outputs of each layer of an CNN-ED model for the input with high signal-to-noise ratio. In the first encoder layer channels 2,3,4,5,7,9 and 10 are not activated for this input. In the second layer, channels 3, 5, 8 and 9 are not activated. All of these channels are activated for a noisy example, shown in Fig.S8.

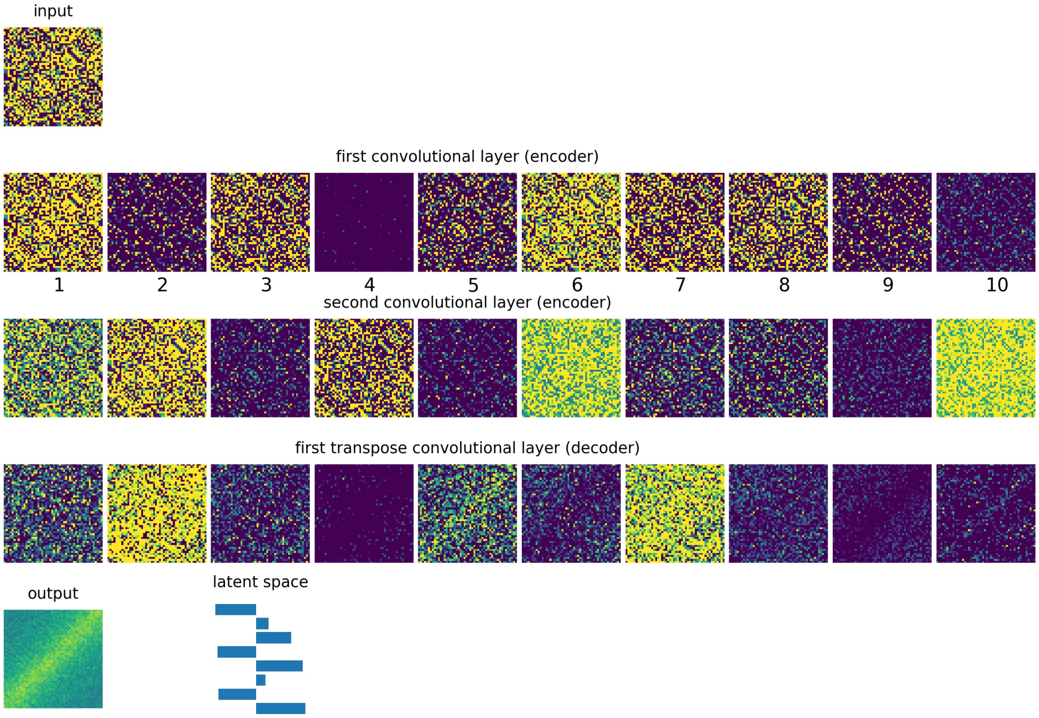

Figure S9. Outputs of each layer of an CNN-ED model for the input with low signal-to-noise ratio.

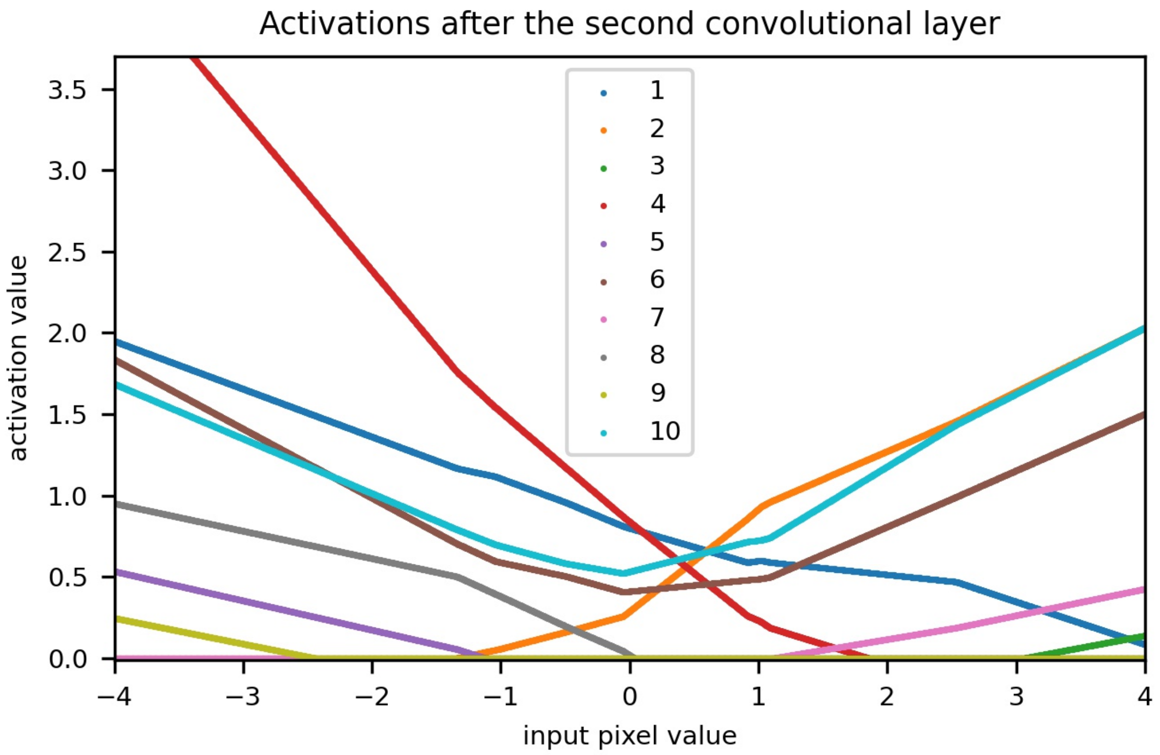

Figure S10. 10 activation functions of the encoder for one of the CNN-ED models.

VI. Model regularization

To avoid over-fitting, several model regularization measures are implemented:

- selecting the simplest model configuration from those that give similar performance on the validation set
- restricting the latent space dimension
- having noise in both inputs and targets
- augmenting the data
- early stopping while training
- combining several models trained with different random initialization into an ensemble

We have also attempted introducing additional weight regularization via increasing the *'weight_decay'* parameter of the Adam optimization algorithm and using dropout channels in the convolutional layers. Both approaches did not improve the model performance on the validation set. Evolution of the training and validation cost functions during a model (10 channels in both convolutional layers, latent space dimension is 20) training with different *'weight_decay'* is shown in Fig. S11. While the increased values of the *'weight_decay'* lead to reduced variance of the model by flattening the tail of the validation cost, they do not reduce the minimum value of it. Similar situation is observed for increasing the dropout probability for the convolutional layers. Thus, we decided not to employ the additional regularization approaches.

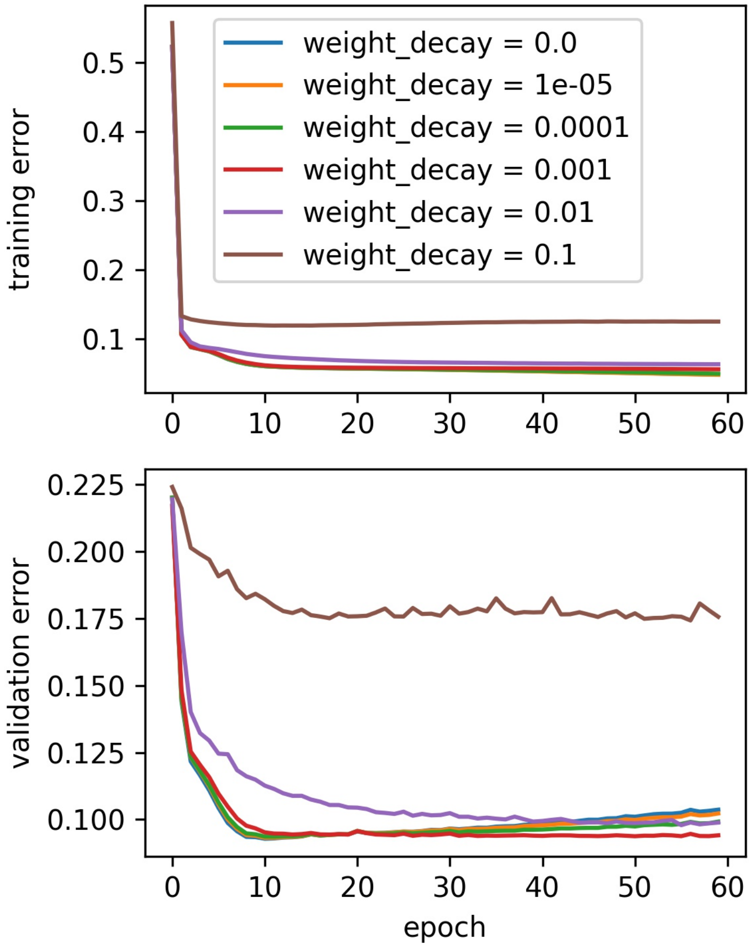

Figure S11. Model training with different *'weight_decay'* parameters.
